# Recalled Coparenting Conflict, Paralysis of Initiative, and Sensitivity to Conflict during Late Adolescence

**DOI:** 10.26502/fjwhd.2644-28840022

**Published:** 2020-05-25

**Authors:** James P McHale, Jean A Talbot, Steven Reisler

**Affiliations:** 1 Department of Psychology, USF St. Petersburg, St. Petersburg, United States; 2 Department of Psychiatry, University of Rochester Medical Center, Rochester, New York; 3 Clinical Psychologist, Nova Southeastern University, Florida, United States

**Keywords:** Paralysis, Late Adolescence

## Abstract

During late adolescence, interpersonal acuity and decisiveness are facilitative of transitions to emerging adulthood. Disruptions in these capacities may be traceable to phenomena evoked by origin family coparental conflict – paralysis of initiative and hypersensitivity to conflict. Documenting such connections can lead to more beneficial interventions for adolescents transitioning into adulthood. The aims of this study were to examine relationships between college freshmen’s reports of coparenting conflict in their origin families and (a) their immobility and indecision when faced with calls to action and (b) their hypersensitivity to signs of inter-adult conflict. Thirty-four freshmen (25 women and 9 men) rated their own coparents’ conflict dynamics and completed (a) a timed perceptual-motor challenge in which quick and deft action was essential to avoid failure; (b) the Rorschach inkblot test; and (c) a judgement task requiring ratings of and predictions about the interpersonal dynamics between unfamiliar adults portrayed in videos coparenting small children. Even controlling for the effects of self-reported depressive symptoms, significant links emerged between greater recalled coparenting conflict in the origin family and longer delays in initiating action in the perceptual-motor challenge; lower active-to-passive responses on the Rorschach; and attributions of more dissonant coparenting behavior in the videotaped family interactions. Results suggest that origin family coparental conflict may show ties to hypersensitivity to conflict and to indecisiveness in the face of calls to action. Implications for theory, research and practice are discussed.

## 1. Introduction

Transitions to young adulthood benefit from interpersonal sensitivity and decisiveness, capacities fostered by emotionally supportive families who encourage instrumentality. Transitions can also be compromised by origin family disturbances [1, 2], including enmeshment [3]. Though depression and anxiety sometimes play a role [4, 5], mechanisms by which family dynamics affect emerging adults’ initiative and assuredness are poorly understood. One trigger is interadult conflicts about the child [6–8], a central facet of “coparenting” [17, 39]. Conflicts between coparents confuse and disorganize children [9], disrupt self-regulatory skills, and hinder relationship development [40]. From a cognitive-contextual perspective [10], the meaning children and adolescents ascribe to their family process is most predictive of individual adaptation. Most studies of coparenting conflict examine under-controlled behavior problems [11], though exposure to conflict is not invariably tied to such problems. One-third of university students describe weekly exposure to coparental conflict during childhood [12], but most show no evidence of clinically concerning impulsivity. However, residuals of family conflict can go unnoticed if studies focus largely on under-control and aggression. Such indicators may have greater salience in understanding the adjustment of male than of female children and adolescents; expressions and perceptions of interpersonal conflict differ in important ways for males and females [13, 14]. This paper addresses two constructs germane to adolescent health. The first, sensitivity to coparental conflict, may have roots in chronic exposure to undermining and triangulating coparenting. Chronic coparental conflict is a threat to family integrity and security [15–17], priming children’s alertness to even negligible signals of dissonance in family groups. Dodge [18–21] linked recurrent aggression in families to youth vigilance to hostile cues and attribution of hostile intent to interpersonal slights from others. Children from families high in husband-wife conflict likewise show heightened sensitivity [22, 23], altered physiological functioning [24], problems self-regulating, and hyper-vigilance to interpersonal conflict [25]. While Dodge finds that children’s hostile attributions portend combative interpersonal behavior, Katz and Gottman’s [26] sensitization theory makes no predictions regarding whether hypervigilance associated with marital conflict is more likely to lead to externalizing or internalizing defenses.

A “paralysis of initiative” residual occupies the opposite end of the spectrum from unarrested impulse. A momentary body-level stasis like fleeting dissociative or hypnagogic states [27], the paralysis is a brief shift in body concept resulting from reduction or change in proprioceptive stimulation while logical thinking is maintained. Herman [28] included helplessness and paralysis of initiative as changes in self-perception central to Complex Post-Traumatic Stress Disorder, though the full CPTSD symptom complex is quite different from the time-restricted, circumscribed bodily phenomenon considered here. In the family literature, paralysis of initiative has seldom been considered, though Bowlby [29] outlined how children whose parents feed them information about their other parent that contradicts the child’s lived experience with that parent face an irresolvable predicament. Even infants show hesitancy, stilling and “freezing” if one parent provides signals that contradict the other [30]. Such momentary body-level paralysis can also be experienced subjectively and may originate when young children are drawn in opposing directions by warring parents [31]. The “tug-of -war” entangling a child offers no remedy when parents compel a choice between their directives. Familiar senses of freezing and of stasis during “moments of truth” may then later be evoked when new unrelated situations call for quick and decisive action. Research examining coparental conflict and adolescent health has never explored paralysis of initiative despite the psychodynamic axiom that unsolvable conflicting feelings, or ambivalence underlie many adolescent mental health issues [32]. Ambivalence is especially insidious if conflict is experienced at young ages; preschool and latency-aged children possess few adaptive resources to cope with conflict, unlike adolescents who use physical distancing strategies (spending time in the haven of friends’ homes, running away) to circumvent inter-adult conflict. Younger children cannot distance, are usually “on site”, and hence often are triangulated into active coparental conflicts [33]. Being forced to ally with one parent against the other stimulates vacillation and ambivalence, an avoidance-avoidance conflict [34]. Some children respond with regressive or externalizing defenses; others internalize, some dissociate. The latter phenomena include incapacitating hesitation when forced to make urgent choices under pressure. To date, there has been virtually no research on young adults’ recollections of coparenting in their families of origin, apart from adults transitioning to parenthood, and hence this study fills an important gap. Based on previous literature suggesting that coparental conflict might be expected to influence (a) hypersensitivity in distinguishing coparental conflict in family situations; and (b) maladaptive diffidence when confronting calls to action, evidence for relevant statistical associations are sought between recalled coparental conflict and two unique and conceptually meaningful sets of adjustment indicators during emerging adulthood – heightened sensitivity to conflict in families about whom the respondent actually knows very little, and signs of inertia when responding to tasks requiring decisiveness.

## 2. Methods

### 2.1 Participants

Participants were 34 college freshmen (25 women and 9 men, M age = 18.2) selected from a larger sample of 237 students enrolled in an introductory psychology course. Twenty-nine were Caucasian, two were African American, one was Asian American, and two were of Hispanic descent. Students were recruited during their initial semester away from home. 50 individuals (thirty women and twenty men) from the larger sample of 237 were initially identified as potential study participants based on (a) having grown up in a home with two identified coparenting figures in residence prior to age twelve; and (b) the nature of their responses to a pre-screen assessing family of origin coparenting conflict. The pre-screen insured a representatively broad range of childhood coparenting experiences. Eighty-five percent of women invited consented to participate, along with 45% of men. Participants and refusers did not differ in reported coparental conflict in the origin family (F 1, 48 = 0.83, ns), or in depressive symptomatology (F 1, 48 = 0.57, ns).

### 2.2 Procedure

The pre-screen was completed during the first week of the transition to college. Follow-up sessions took place in a university lab two months later. A perceptual-motor challenge (Milton Bradley’s “Perfection” ™ game was administered, followed by the Rorschach Inkblot Task, and a videotape rating task (see below). Sessions lasted about one hour. Participants were paid ten dollars. The study received full approval from Clark University’s Internal Review Board (IRB).

### 2.3 Measures

#### 2.3.1 Self-report measures (pre-screening questionnaire)

##### Coparenting conflict in the family of origin

Participants completed items drawn from McHale’s [17] Coparenting Scale, an instrument assessing coparenting behaviors. Students reported how frequently (from 1 - absolutely never, to 7-multiple times daily) during pre-adolescence they encountered disparagement (of father by mother; of mother by father) and undermining of disciplinary interventions (i.e., of father’s interventions by mother; mother’s by father). Indicators constituted a single factor with loadings above .5, and a composite “coparental conflict” index (alpha = .88) summed maternal and paternal disparagement and undermining of discipline items (composite M = 10.03, SD = 3.98, range = 4–21).

##### Depression

To rule out the possibility that poor performance could be more parsimoniously explained by depression, participants completed the Center for Epidemiological Studies Depression Scale (CES-D; [35]). This allowed us to determine whether any significant relations between coparenting conflict and paralysis of initiative fell away once depressive symptomatology was controlled for, or alternately whether depression is a suppressor variable, obscuring associations that might stand out after taking account of depressive symptoms. For this sample, student depression was relatively mild (CES-D M = 4.73, SD = 0.98, range = 1–18).

#### 2.3.2 Laboratory measures

Because the constructs of interest in this study had not heretofore been examined in the literature, we had to devise an approach for estimating them. We developed one set of indicators for the hypersensitivity construct and two for the paralysis of initiative construct.

##### Hypersensitivity to coparental conflict

To measure bias toward perceiving conflict in families, standardized “trigger” stimuli involving brief (30 second) video clips of families at play were used including (a) a “warm-up” tape, depicting a mother, father, and 11-month-old baby interacting together; and (b) two “Test Families” each with 30-month-olds. The warm-up tape contained no conflict; the adults were unusually warm, both with their baby and with one another. It was employed to assure that respondents understood the rating task. Test Family 1 disagreed about undertaking a new activity, with affect that was mixed. They smiled as they bickered, perhaps suggesting they were “jesting” as they sparred. Test Family 2 argued about a loud toy “space” gun. Mother’s affect was solemn as she told father she didn’t like guns. Father gave the gun to the child when mother wasn’t looking, and she then responded negatively. Warm-up and test family footage was drawn from an archive of exemplars of coparental warmth and coparental conflict objectively rated by a panel of trained experts using the Coparenting and Family Rating System [36]. After viewing each clip, participants (1) rated on a scale of 1 (very low) to 9 (very high), (a) overall warmth as well as (b) overall conflict perceived in the clip; and (2) estimated from 0% (never) to 100% (all the time) what percentage of time during everyday life parents in the video (a) said disparaging things about one another to the child, and (b) undermined one another’s disciplinary efforts. In other words, based on only 30 seconds of information they predicted how often the coparenting conflict experienced in their own families transpired in families about whom they knew virtually nothing. The “prediction” scores (mother-disparage; mother undermine; father disparage; father undermine) were summed forming a single index (from 0 to 400) indicating expectation of chronicity of coparental Conflict at Home. Higher scores reflected an expectancy of more pervasive conflict. Descriptive data are presented in Table 1. We anticipated a significant positive association between higher recalled coparental conflict during childhood and higher “conflict” ratings for Test families – particularly Test family 1 (who showed mixed affect) - reasoning that conflict histories relevant to coparenting would sensitize respondents to ambiguous fights. We also hypothesized a significant association between high conflict histories and predictions of more chronic inter-parental conflict in the day-to-day lives of the two Test families.

##### Paralysis of initiative

To provoke temporary immobility, we chose a task involving time urgency. The task was the Milton Bradley ™ game “Perfection” in which a collection of small geometric shapes must be placed into matching puzzle slots before a timer sounds. Correct placement of all pieces within the allotted time prevents the game board from popping up and abruptly dislodging the pieces placed by the player. Rapid response is thus adaptive. We expected that students prone to stasis and paralysis of initiative would take longer to act and so show a greater latency to place the first puzzle piece. For this sample, mean time to place the first piece was 4.32 seconds, SD = 3.65, range = 2–21 seconds. A second index was a task long known to evoke uncertainty -- the Rorschach Inkblot Test. Respondents confronted a situation of uncertainty and were pressed to make judgments. We looked to a particular set of summary scores yielded by formal coding of the Rorschach, the “active-to-passive” ratios both for the complete record (total Active: Passive ratio for the sample 204:106; see Table 1) and, more specifically, for the Human Movement responses (total Ma: Mp for the sample, 80:61; Table 2). Active responses in response to Rorschach stimuli involve some type of action or movement, such as “two clowns slapping hands”. Passive responses are devoid of action, such as “two girls facing each other”.

The significance of the active/passive ratios was first discussed by Rorschach, who drew a distinction between “flexor” (inward movement) and extensor (outward movement) dimensions. There is a fascinating literature on this distinction, including findings that extensors are more resistant to contradiction, while flexors are more likely to succumb. Based on prior research with related phenomena [37], we believed this index had relevance to the immobility and paralysis construct we sought to capture, reasoning that a lower ratio of active-to-passive responses might serve as a “proxy” indexing an embodied inhibition that hampered strong and decisive engagement.

## 3. Results

### 3.1 Coparenting conflict and hypersensitivity to conflict

Analyses suggested that construals of the videotaped family interactions may have been colored by participants’ histories with family conflict, though not entirely in the manner anticipated. We predicted significant associations between higher recalled coparental conflict and both higher ratings of coparenting conflict in the first “Test” family (where conflict was intermixed with jesting), and predictions of greater Conflict at Home. Data did not support this hypothesis; there were no associations between recalled coparental conflict and either the overall ratings of conflict detected during video interactions (r = −.26, ns), or predictions about coparental Conflict at Home (r = .04, ns). Quite unexpectedly, in fact, exactly the opposite “skew” was found. That is – the more chronic the recalled coparenting conflict in the origin family, the higher respondents rated Test Family 1’s warmth (r = .58, p < .05). Though counterintuitive at first glance, recall that the coparenting partners in this video showed ambiguous affect -- cutting barbs offered jokingly. Perhaps perceiving gleams of warmth during an otherwise antagonistic interaction reflected a bias among children of conflict to be attentive to any hint of positive coparental connection, whereas those less accustomed to coparental conflict were less prone to construe such behavior as indicative of warmth. Findings for the second Test Family 2, in which the parents unquestionably engaged in a negative exchange, were in line with predictions. There were significant associations between recalled coparenting conflict and both participant ratings of high conflict for the couple on video (r = .45, p < .05), and predictions of greater Conflict at Home in the lives of these unknown individuals (r = .35, p < .05). No association was found between recalled coparenting conflict and Test Family 2’s warmth (r = −.16, ns). Taken together, data provide beginning evidence that young adults’ construals of present-day family conflict, even when based on very limited evidence, can be meaningfully linked to recollections of coparenting distress in their own origin families.

### 3.2 Coparenting conflict and paralysis of initiative

Analyses substantiated a significant association between recalled coparenting conflict and latency to place an initial puzzle piece in the Perfection game. Specifically, there was a significant positive relationship between reports of having been exposed to more chronic coparental disparagement and undermining during childhood and time it took to act in a situation calling for quick and decisive action (r = .36, p < .05). This association not only persisted, but was mildly strengthened, after partialling out depressive symptomatology (pr = .39, p < .05). These findings indicate a linkage between recalled origin family coparental conflict and maladaptive hesitancy during a call to quick action. Evidence was also found for a link between origin family coparental conflict and the active to passive ratio in the Rorschach record. Specifically, greater recalled coparental conflict was associated with a lower proportion of active responses in the Rorschach protocol (r = −.45, p < .05). This pattern was even clearer for just Human Movement responses (r = −.55, p < .05). Controlling for current depression did not affect the significance vs. non-significance of these findings (prs of −.43 and −.52, p < .05, for the two ratios, respectively). Given the relatively small sample size, exploratory nature of proxies used to estimate the paralysis construct, and 2-month time lapse between initial reports of family distress and ensuing laboratory assessments, these preliminary findings hint at links between coparental conflict and the arresting sense of uncertainty and immobility of interest in this study.

## 4. Discussion

This study traced linkages between recollections of coparental conflict and two individual difference constructs relevant to adolescent adjustment -- sensitivity to inter-adult conflict and paralysis of initiative during calls to action. Both may be pivotal and predictive of adjustment during developmental transition points. Preliminary findings support the contention that coparental conflict may heighten vigilance to signs of hostility and conflict, and produce a sense of temporary psychic immobility, inhibition from taking decisive action or paralysis of initiative. This latter construct is a new, complex one, so findings are primarily of heuristic value as they reflect data collected in a laboratory context. Equally, we speculate patterning of findings may be even more striking if sensitivity to conflict and calls to action are studied in a more salient social context; some of Dodge’s most compelling data involve observed social interactions among peers. In the current study hostility dwelled only on family films; vigilance and hypersensitivity may be even more conspicuous in circumstances involving intimates. Similarly, inhibition to decisive action was sampled only via laboratory tasks; immobility may be most strongly in evidence within social situations. Evaluating the phenomena examined in this study within pertinent social contexts invites future inquiry. Study participants no longer lived at home, so it is possible to construe the phenomena of interest as enduring traits or styles of responding. Indeed, vigilance to conflict could help paralysis of initiative persevere. Children who experience chronic coparental conflict learn to view the interpersonal world with an eye toward discord. If unfamiliar present-day situations contain discord, less secure individuals may engage such settings cautiously and passively. Paralysis of initiative could stem from hesitance about acting in a hostile environment. This explanation involves a high level of cognitive mediation, whereas the phenomenology of those who report feeling “paralyzed” indicates that the experience is a thoroughly embodied one. Nonetheless, it may be worth further examination.

Another possibility -- that the ephemeral paralysis might be a dissociative-spectrum process instigated by perceived threat to interpersonal security -- is intriguing. Many children with contentious coparents identify threat even in seemingly unremarkable social situations that possess only low or mild interpersonal discord. Within a trauma framework, brief reductions in sensory input from the body (or failure to attend to such) occur, even as awareness and logical thinking are maintained. The appeal of this explanation is its link to an established conceptual framework on traumatic experience, lack of reliance on cognitive processing, and consonance with the phenomenology of feeling “frozen” and unable to take decisive action despite sufficient cognitive resources. It does not explain, however, why immobility and momentary paralysis would surface in less extraordinary situations where a call to immediate action is not socially embedded. Each of these interpretations is consistent with an emotional security hypothesis and with impact of insecure attachments - particularly disorganized attachments - on inhibiting choiceful behavior and exploratory competence. Other factors not included in the study may also be pertinent. First, we targeted only one family process - coparental conflict - not respondents’ dyadic relationships with each individual parent. Relatedly, even if subsequent studies replicate links between coparental conflict and paralysis of initiative, it is unclear whether immobility and indecision stem from an embodied sense of being pulled in opposing directions (as we contend), having identified with a same-sex parent chronically portrayed by the other parent as undependable and incompetent, a more generalized fear response, or some other intrapsychic processes. Second, indicators selected for study may not have been ideal choices to approximate the constructs of interest, although the overall patterning of results -- in virtually every case pointing in the hypothesized direction -- from a high functioning sample, is quite heartening. Results did not simply reflect more depressed students remembering more volatile home environments and performing more poorly on lab tasks; other relevant personality dimensions (introversion, intellectualizing defenses, lack of surgency) warrant study in future work. It is also important to establish whether the paralysis phenomenon is applicable only in select samples (such as highly achieving individuals), or whether it has parallels elsewhere. Other relevant concerns include the comparatively small sample, comprised primarily of adolescent females, which prohibited gender difference analyses. Yet this sample composition renders findings worthy of note. Prior research implies that coparental conflict affects males most adversely; it is almost always boys who show the greatest disruptions in functioning. One reason such studies may not uncover correlates of coparental conflict among girls is that outcomes typically examined (externalizing-spectrum problems) fail to adequately assess relevant behaviors among youth who respond to inter-adult sparring and triangulating by arresting, rather than discharging impulses inappropriately.

In summary, this report highlights two understudied constructs perhaps inextricably tied to chronic coparental conflict. Subsequent research with more refined measurement approaches is needed, and may further extend these promising results obtained with a small sample of well adjusted, non-clinically referred college students. It is also possible super-ordinate constructs such as generalized anxiety or ego over-control [38] will prove better fits for the data. In either case, the current work invites further investigation into these important after-effects of childhood coparental distress and may be of pragmatic use to those working with adolescents and young adults during times of transition.

## Figures and Tables

**Table 1:** Means and Standard Deviations for Students ratings of the two “Test” Family tapes.

Test Family	Warmth	Conflict	Conflict at Home
Test Family 1	3.48 (0.97)	6.52 (1.06)	173.24 (71.88)
Test Family 2	3.61 (0.89)	5.61 (1.22)	155.58 (63.06)

**Table 2:** Summary of Relevant Rorschach Protocol Responses.

Card #	# of Human Movement	# of Animal Movement	# of Inanimate Movement	#Ma	#Mp	# of Active	# of Passive	# of COP
I	12	10	0	7	5	14	8	4
II	23	8	7	18	5	30	8	17
III	27	10	3	19	8	26	14	20
IV	14	8	2	3	11	8	16	0
V	4	10	1	1	3	12	3	1
VI	3	9	4	1	2	10	6	0
VII	28	5	5	12	16	19	19	12
VIII	7	32	4	4	3	34	9	1
IX	8	10	7	6	2	18	7	3
X	15	24	10	9	6	33	16	8
	Total # of M	Total # of FM	Total # of m	Total Ma:Mp		Total Active :Passive		Total # of COP
	141	126	43	80:61		204:106		66

Grand Summary (for all 34 protocols combined)
